# Backpack Load Carriage Affects Motor and Sensory Responses of the Median Nerve

**DOI:** 10.1093/milmed/usaf459

**Published:** 2025-09-22

**Authors:** Jennifer L Hein, Katherine Saul, Deanna J Schmidt

**Affiliations:** Department of Kinesiology, California State University San Marcos, San Marcos, CA 92096, United States; Department of Mechanical and Aerospace Engineering, North Carolina State University, Raleigh, NC 27606, United States; Department of Kinesiology, California State University San Marcos, San Marcos, CA 92096, United States

## Abstract

**Introduction:**

Use of the upper limbs is often necessary for military and firefighter personnel who carry backpacks. Backpack straps can compress the brachial plexus nerves of the upper limb. It is known that carrying a backpack can lead to rucksack palsy, but it is unknown if effects of upper limb nerve compression from carrying a heavy backpack can be demonstrated after a single session of backpack use. Our study aimed to investigate the short-term effects of backpack carriage on upper limb nerve conduction.

**Materials and Methods:**

Thirty-six participants including 18 female (mean ± SD: age 24.3 ± 7.6 years; height 168.8 ± 9.4 cm; mass 73.1 ± 16.6 kg; BMI 25.4 ± 4.5 kg/m^2^) and 18 male (24.1 ± 5.8 years; 178.2 ± 9.4 cm; 80.2 ± 11.7 kg; BMI 25.0 ± 4.3 kg/m^2^) were recruited as a convenience sample and assessed by nerve conduction study (NCS) of the median nerve on the dominant upper limb. Skeletal muscle mass (SMM) and body mass index (BMI) were evaluated using bioelectric impedance. Nerve conduction study measurements were taken before (PRE), after 20 minutes of walking and while still wearing a military-style large frame backpack with hip belt loaded to 30% bodyweight (POST), and immediately after removing the backpack (DOFF). We analyzed comparisons statistically using mixed factor analysis of variance (ANOVA) with significance level of *P* < .05.

**Results:**

Motor nerve action potential amplitude significantly decreased when stimulation was at the axilla from PRE to POST (*P* = .025) and PRE to DOFF (*P* = .012). Motor nerve action potential latency was significantly increased PRE to POST and PRE to DOFF with stimulation at the elbow (*P* = .029 and *P* = .030, respectively). Latency was significantly longer for males as compared to females (*P* ≤ .008). Sensory nerve action potential amplitude decreased significantly between PRE and POST (*P* = .007). Significant correlation was determined between amount of SMM and percent difference PRE to POST in motor nerve action potential amplitude (*r *= 0.438, *P* < .01). Participants with lower SMM demonstrated greater difference in motor nerve action potential after backpack carriage with POST measurements lower than PRE measurements. Body mass index was significantly correlated with sensory baseline-to-peak amplitude percent difference PRE to POST (*r* = 0.428, *P* < .01) indicating that those with lower BMI had a larger negative impact on sensory nerve response after backpack carriage.

**Conclusion:**

The results of this study reveal that walking for 20 minutes while carrying a 30% bodyweight backpack resulted in an increase in upper limb motor nerve latency and decrease in sensory and motor nerve action potential amplitude. The NCS findings indicate that SMM may have a protective effect and was therefore beneficial to maintaining upper limb nerve conduction after backpack carriage while lower BMI was a risk factor for reduced sensory nerve conduction. The demonstrated deficits in nerve conduction after backpack carriage could have implications for use of hands, especially the thumb, index, and middle finger, as they are innervated by the median nerve.

## INTRODUCTION

Military personnel and wildland firefighters often need to do tasks with their hands, at times accurately and swiftly.^1–3^ Manual tasks can include carrying equipment, deploying syringes, maneuvering tools, operating communication devices, and handling ammunition or weaponry.^4–6^ These same personnel often carry backpack loads of 30% bodyweight or more.^7–11^ Loads applied by backpack shoulder straps can put pressure on the brachial plexus nerves and constrict blood flow to the upper limbs.^1^^,^^12^^,^^13^ These heavy loads carried by backpack straps can potentially cause pain and injury, as well as affect use of the arms.^14–18^ For these reasons, it is important to understand if upper limb motor and sensory nerve response is impacted by carrying a backpack.

Carrying a heavy backpack can lead to backpack or rucksack palsy, especially for those whose occupation requires repeated backpack use.^16^^,^^19–21^ Incidence rate of backpack palsy has been reported as up to 5.6 cases per 10,000 military members.^16^^,^^22^ Risk factors for backpack palsy include weight of load carried in the pack, duration of load carriage, and speed of locomotion.^16^^,^^22^^,^^23^ Amount of muscle tissue in the shoulder area and body mass index (BMI) have also been correlated to backpack palsy incidence, with lower BMI corresponding to higher incidence of backpack palsy.^15^^,^^22^ Effects of nerve compression from backpack carriage include increased muscle fatigue and muscle weakness.^16^^,^^17^^,^^23^ Backpack palsy can result in lack of sensation, tingling, pain, and muscle weakness in the arms and hands.^16^^,^^21^ Several studies have reported that removing the backpack can result in reduction of pain, but that muscle weakness and sensory deficits may persist.^16^^,^^19^^,^^21^^,^^24^ Additionally, in one report, over 20% of individuals still reported symptoms of backpack palsy at a 4.5-year follow-up.^21^ Yet, the mechanisms that underlie backpack palsy remain unclear. It is also unknown if short-term effects on nerve conduction are present after walking with a heavy backpack load or if changes in upper limb nerve conduction only occur after repeated backpack carriage. Improved understanding of the effects of nerve compression from backpack straps could lead to possible ways to improve symptoms, reduce injury, and allow military and firefighter personnel to maintain optimal use of their hands. Studying nerve conduction in the upper limbs is a significant step toward understanding readiness after backpack carriage for military and firefighter personnel as slowed motor response or loss of sensation on the fingers may increase risk of injury and slow completion of job-related tasks. Upper limb nerve conduction study (NCS) after backpack carriage could provide increased knowledge of risk factors for nerve compression and lend insight toward developing guidelines to reduce the rate of nerve compression symptoms and backpack palsy.

Our aim was to determine whether nerve conduction in the median nerve is affected in the short term after walking with a backpack load. The median nerve is a mixed motor and sensory nerve in the upper limb.^25^ We hypothesized that because of compression of the nerves of the brachial plexus, the action potential amplitude would be decreased for motor and sensory nerve conduction in the median nerve. We also hypothesized that latency of the motor nerve action potential would increase, indicating a delay in timing of the action potential after walking with a 30% bodyweight load in a backpack. Additionally, we expected the velocity of conduction in the median nerve to decrease after carrying a backpack load. We used repeated measures of upper limb NCS to test these hypotheses in male and female participants.

## METHODS

Thirty-six participants including 18 female (mean ± SD: age 24.3 ± 7.6 years; height 168.8 ± 9.2 cm; mass 73.1 ± 16.6 kg) and 18 male (24.1 ± 5.8 years; 179.3 ± 6.8 cm; 80.2 ± 11.7 kg) were recruited as a convenience sample from the university and surrounding community. Six participants (1 female) had current or prior military experience. All participants provided informed written consent to protocols approved by the Institutional Review Board at California State University San Marcos (IRB# 1601172-1, approved June 5, 2020). All participants self-­reported as right hand dominant. Statistical software G*Power Analysis (Universitat Keil, Germany) was used to determine that 28 participants were needed to power the analysis of variance (ANOVA) comparisons estimating a medium effect size and level of significance of *P* < .05. Participants were excluded for known cardiovascular, respiratory, or musculoskeletal disease. Potential participants were excluded for back, shoulder, or upper limb injury or surgery within the previous 5 years. We instructed participants to abstain from vigorous physical activity lasting over 1 hour within 48 hours before testing. We also asked participants to abstain from eating heavy meals, caffeine, and nicotine within 3 hours before the trials.

Bioelectric impedance (InBody, United States) was used to assess body mass, BMI, and skeletal muscle mass (SMM) of each participant. Participants wore comfortable athletic clothing and footwear supportive for walking. A military-style backpack with external frames, a thoracic strap, and a hip belt was used for the study (United States Marine Corps (USMC) pack System: 8465-01-598-7693 Main Bag: 8465-01-600-7911, Eagle Industries). We properly fit each participant in the backpack per the Program Manager Infantry Combat Equipment.^26^ Participants walked on a treadmill (Bertec Corporation) at 1.1 m/s for 20 minutes carrying a load of 30% bodyweight (BW) in a military-style backpack. The 30% BW load was selected as this is the recommended assault load for the Marine Corp and similar to the load level carried by wildland firefighters.^3^^,^^27^ Walking speed was based on previous research reporting a mean self-selected speed of 1.1 m/s while carrying a load.^28^ Participants were attached to an overhead gantry for safety by non-weight bearing cables at the backpack’s donning straps while on the treadmill. Participants were instructed to keep their arms free swinging and not to hold on to the backpack straps while walking.

Measurements of upper limb nerve conduction were ­performed immediately before each walking trial before the participant donned the backpack (PRE), immediately after the walking trial with the backpack on (POST), and immediately after the backpack was removed (DOFF).

Sensory and motor nerve action potential amplitude, velocity, and peak latency were recorded for the dominant upper limb using surface electrodes. The median nerve was stimulated (Neurodiagnostic System, Natus Inc.) at the wrist, elbow, and axilla for motor nerve action potential measurements of the nerve conduction studies. Two researchers completed training for nerve conduction studies and performed all nerve stimulation measurements. Electrical stimulation was delivered with supramaximal stimulation 8 cm proximal to the active electrode for the wrist location motor stimulation. Median nerve stimulation points were located at the elbow and axilla for each participant and the distance from the stimulation point to the recording electrode was measured and documented. Stimulation was 0.2 ms duration. For the motor nerve stimulation, the active recording electrode was placed on abductor pollicis brevis. For sensory nerve stimulation, the active recording electrode was placed on the skin of the most proximal segment of the index finger. Sensory nerve action potentials were achieved by stimulating at the wrist. Stimulation was 0.1 ms in duration and occurred 14 cm proximal to the active electrode for sensory nerve conduction measurements. Sensory NCS was applied antidromically. Both baseline-to-peak (NP) amplitude and peak-to-peak (PP) amplitude were recorded for sensory nerve recordings. Electrodes for nerve conduction measurements were outlined with a marker to ensure the same placement for PRE, POST and DOFF measurements. Stimulation location points for the wrist, elbow, and axilla were also marked. Electrodes were removed before participants walked on the treadmill.

Data were analyzed using Microsoft Excel and SPSS (IBM Inc.). Means and SDs for all measures were computed. Mixed factor ANOVA was used to evaluate differences in nerve response among timepoints and between sexes. We completed Bonferroni post-hoc tests to determine significant comparisons. We used partial eta squared to evaluate effect size. Correlations between SMM, BMI, and percent difference between the nerve conduction measures PRE to POST and PRE to DOFF were performed to determine correlation coefficient, r. Percent difference was computed as (POST-PRE)/PRE, for example, such that a negative percent difference indicated a lower POST value compared to PRE value. *P* values were determined for correlations using a correlation table. We chose a standard level of statistical significance at *P* < .05.

## RESULTS

### Motor Nerve Conduction

Motor nerve action potential amplitude with stimulation at the axilla showed a main effect for timepoint (*F* = 6.414, *P* = .006, **Table 1**). Specifically, the motor nerve action potential amplitude with stimulation at the axilla significantly decreased from PRE to POST (*P* = .025, 8.4% decrease) and from PRE to DOFF (*P* = .012, 5.9% decrease). Stimulation at the wrist and elbow produced no significant changes in motor nerve action potential amplitude when comparing PRE, POST, and DOFF timepoints (**Table 1**). The motor nerve data with axilla stimulation point supported our hypothesis that action potential amplitude would be decreased after backpack carriage. Peak latency of motor nerve conduction had a significant main effect of timepoint for stimulation at the elbow (*F* = 6.452, *P* = .007, **Table 1**). Motor nerve action potential latency was significantly increased from PRE to POST and from PRE to DOFF with nerve stimulation at the elbow (*P* = .029 and *P* = .030, respectively). Motor nerve latency also showed significant increase PRE to DOFF when the median nerve was stimulated at the axilla (*P* = .008). Therefore, data recorded with stimulation at the elbow and axilla statistically supported our hypothesis that nerve latency would increase after backpack carriage. Conduction velocity did not differ significantly from PRE to POST or DOFF timepoints regardless of stimulation location contrary to our hypothesis (**Table 1**).

**Table 1:** Median Nerve Motor Conduction Measures Resulting From Nerve Stimulation

Measure	Stimulation site	PRE	POST	DOFF
**Amplitude (mV)**	Wrist	9.88 (3.12)	9.30 (3.42)	9.37 (3.19)
	Elbow	9.45 (2.94)	8.65 (2.97)	9.32 (2.97)
	Axilla	12.24 (2.42)	11.21 (2.64)^a^	11.52 (2.22)^a^
**Latency (ms)**	Wrist	3.27 (0.56)	3.34 (0.56)	3.31 (0.56)
	Elbow	7.52 (0.69)	7.63 (0.82)^a^	7.64 (0.80)^a^
	Axilla	9.43 (0.87)	9.51 (0.97)	9.57 (0.94)^a^
**Velocity (m/s)**	Wrist	25.22 (4.77)	24.64 (4.28)	24.92 (4.88)
	Elbow	57.51 (5.49)	56.10 (6.33)	56.44 (4.50)
	Axilla	68.77 (7.83)	69.08 (8.27)	67.28 (7.74)

Values are mean (± SD); *n* = 36; 18 female, 18 male. Comparisons made among timepoints by mixed-factor analysis of variance and Bonferroni post hoc testing.

a Significant difference, *P* < .05, from PRE timepoint measurement.

Abbreviations: DOFF, after removing the backpack; POST, after carrying 30% bodyweight backpack walking for 20 min with backpack on; PRE, before carrying backpack.

There was a significant effect of sex for motor nerve latency when the median nerve was stimulated at the elbow (*F* = 9.017, *P* = .005) and for latency with stimulation at the axilla (*F* = 7.849, *P* = .008). Male participants demonstrated significantly longer motor nerve latency than female participants. There was a significant interaction effect of sex and latency when stimulation was at the elbow (*F* = 4.860, *P* = .019). There were no differences between male and female participants for motor nerve action potential amplitude when the median nerve was stimulated.

### Sensory Nerve Conduction

**Figure 1.**
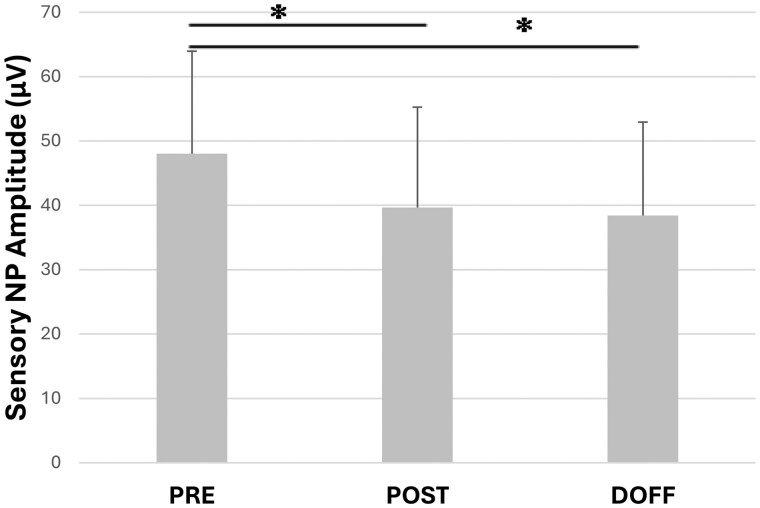
Mean sensory baseline-to-peak (NP) action potential amplitude measured by nerve conduction study (*n* = 36; 18 female, 18 male) at 3 timepoints including: before donning the backpack (PRE), immediately after walking for 20 minutes with 30% bodyweight backpack on (POST), and immediately after the backpack was removed (DOFF). Error bars are 1 SD. *Indicates a statistically significant difference, *P* = .007 PRE to POST and *P* < .001 PRE to DOFF, between 2 timepoints connected by a horizontal line.

Sensory response of the median nerve was affected by carrying a backpack load. There was a main effect for timepoint of measurement on sensory nerve action potential amplitude (*F* = 10.556, *P* < .001). Baseline-to-peak (NP) sensory amplitude decreased significantly between PRE and POST timepoints (*P* = .007; **Figure 1**). The mean NP amplitude of the sensory response decreased 17% from PRE to POST. DOFF sensory NP amplitude was also significantly less than PRE sensory amplitude (*P* < .001; **Figure 1**). The mean sensory NP amplitude decreased 20% from PRE to DOFF timepoints. There was no difference between sexes for decrease in sensory NP amplitude. Sensory nerve latency was not affected by backpack carriage as PRE, POST, and DOFF peak latency averaged 3.3 ms. Similarly, velocity of sensory nerve conduction did not change after backpack carriage, with a PRE average of 55.3 m/s and POST and DOFF averaging 55.4 m/s. Our hypothesis that sensory nerve action potential amplitude would be decreased after backpack carriage was clearly supported by the statistical comparisons with p values <0.007, but the null hypotheses could not be rejected for sensory nerve latency and conduction velocity measures.

### Correlations

For the motor nerve conduction, significant correlations were found between SMM and percent difference in latency PRE to POST (*r* = 0.402, *P* < .025, **Table 2**) and POST to DOFF (*r* = 0.339, *P* < .05) when stimulation was at the elbow. Significant positive correlations were also demonstrated between SMM and motor nerve action potential amplitude percent difference PRE to POST (*r* = 0.438, *P* < .01) and PRE to DOFF (*r* = 0.462, *P* < .01) when axilla level stimulation was performed. The correlation demonstrated that participants with less SMM had larger and more negative difference in motor nerve action potential between POST and PRE or DOFF and PRE timepoints (**Table 2**). Average SMM was 27.8 ± 4.2kg and 37.8 ± 5.1 kg for female and male participants, respectively. For sensory nerve response, a significant correlation was observed between SMM and sensory peak-to-peak (PP) amplitude percent difference from PRE to DOFF (*r* = 0.433, *P* < .01). Additionally, BMI was significantly correlated with sensory PP amplitude percent difference PRE to DOFF (*r* = 0.344, *P* < .05) and with sensory NP amplitude percent difference PRE to POST (*P* < .01, **Table 2**). The correlation indicated that those with lower BMI had a larger negative impact on sensory nerve response after backpack carriage. Average BMI was 25.4 ± 4.5 kg/m^2^ for female participants and 25.0 ± 4.3 kg/m^2^ for male participants.

**Table 2.** Significant Correlations (*n* = 36) for Percent Difference Between 2 Timepoints Surrounding 20 Minutes of Walking With 30% Bodyweight Backpack Carriage in Motor Nerve Response and Sensory Action Potential Amplitude in the Median Nerve and Skeletal Muscle Mass or Body Mass Index for Each Participant

Measure	% Difference	Stimulation Site	Time Points	Mean % Difference	*r*	*P*
	***Motor response***					
**Skeletal muscle mass**	Latency	Elbow	PRE to POST	1.4	0.402	<0.025^a^
**Skeletal muscle mass**	Latency	Elbow	PRE to DOFF	1.5	0.339	<0.05^a^
**Skeletal muscle mass**	Amplitude	Axilla	PRE to POST	−6.0	0.438	<0.01^a^
**Skeletal muscle mass**	Amplitude	Axilla	PRE to DOFF	−3.9	0.462	<0.01^a^
	***Sensory response***					
**Skeletal muscle mass**	PP amplitude	Wrist	PRE to DOFF	−14.0	0.433	<0.01^a^
**BMI**	NP amplitude	Wrist	PRE to POST	−10.3	0.428	<0.01^a^
**BMI**	PP amplitude	Wrist	PRE to DOFF	−14.0	0.344	<0.05^a^

Abbreviations: BMI, body mass index; DOFF, after removing the backpack; POST, after carrying 30% bodyweight backpack walking for 20 min with backpack on; PP, peak-to-peak amplitude; PRE, before carrying backpack; *r*, correlation coefficient.

a Significant correlation with *P* < .05.

## DISCUSSION

This work reveals possible mechanisms underlying reduced upper limb function associated with compression caused by backpack straps through examination of upper limb nerve conduction. The aim of this research was to investigate the short-term effects on the upper limbs after carrying a 30% bodyweight backpack. The results reveal that compression by the backpack shoulder straps had a significant effect on nerve conduction. To our knowledge, this is the first study to present data demonstrating acute deficits in nerve conduction related to backpack carriage. Sensory and motor nerve conduction for the median nerve were decreased after 20 minutes of walking while carrying a heavy backpack. There was significantly greater motor nerve peak latency in male as compared to female participants. The hypotheses of decreased action potential amplitude and increased latency were supported by the measurements of median nerve conduction following heavy backpack carriage. However, conduction velocity in the median nerve was unchanged after carrying a backpack, contrary to our hypothesis. The decreased nerve conduction action potential amplitude and increased latency could potentially affect use of the hands and contribute to muscle weakness or delay in muscle contraction immediately following backpack carriage. The amplitude of the sensory response on the second finger continued to decrease after backpack removal with DOFF timepoint measurements having the lowest mean amplitude and a 20% decrease from the PRE timepoint.

Several factors are related to risk for backpack palsy and risk for musculoskeletal injury, including the amount of load carried, duration of load carriage, sex, and physical fitness.^16^^,^^17^^,^^23^^,^^29^ Risk related to amount of backpack load can vary by occupation, such as first responder, military and firefighter, and role within an occupation. The absolute weight of the backpack at the onset of backpack palsy symptoms has been noted to affect the severity of the resulting muscle strength deficit.^21^ The 30% bodyweight load carried in this study averaged 22 kg for females and 24 kg for males. Previous research on backpack carriage cites a recommended maximum load of 23.1 kg or 40% bodyweight,^30^ however, military members often carry backpack loads that can be from 22 kg to above 45 kg.^17^ The 30% bodyweight backpack carried in the current research is similar to the load carried by wildland firefighters who may need to use their arms and hands to maneuver chainsaws and hand tools.^3^ Military backpack load is often determined by role or unit, so it is possible that smaller individuals, including female personnel, may carry a higher percentage of their bodyweight.^31^^,^^32^ The amount of time under backpack load may also play a role in risk of nerve injury. The time that participants carried the load in the current study is much shorter than duration of backpack carriage for military personnel who hike for many hours and wildland firefighters who work 12-hour shifts.^3^^,^^12^ Therefore, it is notable that significant nerve conduction changes were measured after walking with a 30% bodyweight load for a duration of 20 minutes. The present findings suggest that even within the first 20 minutes of load carriage, compression of the brachial plexus by backpack shoulder straps results in significant nerve conduction deficits.

Previous studies have reported relationships between BMI, hypertrophy of muscle, and incidence of backpack palsy.^16^^,^^22^ Lean body mass was also recently found to be correlated to performance times for load carriage trials of 400 m and 3,200 m.^33^ Fallowfield et al.^34^ reported decreased lower limb neuromuscular response after backpack carriage for male participants who weigh less as they experienced higher heart rate and decreased ability to perform a vertical jump. In comparison, we found a correlation between SMM and both latency and amplitude of the upper limb motor nerve response. We also determined a significant correlation with sensory nerve response deficit and BMI that illustrated those with low BMI had greater difference in sensory nerve response after load carriage. The present data extend the load carriage related correlations with body composition to support that amount of SMM and BMI may play a role in upper limb nerve conduction after backpack carriage. Specifically, those individuals with higher SMM also had less negative percent differences between PRE and POST measurements in both motor nerve latency and action potential amplitude and in PRE and DOFF sensory nerve action potential amplitude. These results add to those reported by Kim and Kim^22^ stating the Korean soldiers with mean age of 20.6 years who were symptomatic for backpack palsy had a lower mean BMI, 21.7 ± 2.7 kg/m^2^, than the BMI of a standard company at 23.2 ± 4.0 kg/m^2^. It should be noted that the present study used a convenience sample of participants with higher average age, higher BMI, and less military experience than those in the Kim and Kim^22^ study, but our results similarly support that lower BMI may contribute to increased risk for brachial plexus compression under the backpack shoulder straps. Our findings also support the expectations of research conducted retrospectively on Finnish soldiers as body composition was a factor in nerve conduction response.^35^ All participants in our study wore an external-frame backpack with a buckled hip belt and chest strap. The required buckling of all belts could have biased the data in a way that is different from retrospective studies of military members who may not always buckle the hip belt or chest strap. Use of a hip belt has been shown to decrease incidence of compression neuropathy.^35^ The current results suggest that SMM had a protective effect and was therefore beneficial to maintaining upper limb nerve conduction after backpack carriage while lower BMI was a risk factor for reduced sensory nerve conduction.

Several limitations to this study are worth consideration when interpreting results. First, only the dominant upper limb was measured, and it is possible that the non-dominant limb may have different results.^1^ One previous investigation found no significant difference between dominant and nondominant arm when examining fatigue in shoulder muscles after backpack carriage,^36^ while another recent study determined that grip strength for the nondominant arm was more affected after running with load.^37^ Based on differences in previous research and our findings, future research is needed to test nerve conduction and its effects for both upper limbs. Also, nerve measurements in the present study were taken immediately after load carriage and immediately after dropping the backpack and hence represent acute short-term changes in nerve conduction. The results cannot be extrapolated to longer times after backpack removal. Additionally, the effects of backpack straps were studied only on the median nerve in this research. It is possible that other nerves in the upper limb, such as the long thoracic nerve, could also be affected by backpack carriage.^20^ Further exploration of upper limb nerve response after longer duration, heavier backpack carriage, and in other brachial plexus nerves is needed to address these limitations. Future research will broaden the understanding of the relationship between short-term nerve compression, functional deficits, and backpack palsy.

Females tend to be at higher risk than males for musculoskeletal injuries related to backpack carriage because of the loads being a higher percentage of bodyweight as compared to males.^29^^,^^32^ However, physical fitness was found to be a mitigating factor in injury risk for both males and females.^29^ One previous study also reported no significant differences found in muscle activation and gait pattern between males and females when walking with a backpack load of 10%, 20%, and 30% bodyweight.^10^ In contrast, Hoolihan et al.^38^ reported female participants to have greater change in lower body kinematics as compared to males which suggests that carrying load may affect the sexes differently. The present study adds to the comparison between sexes during backpack carriage by demonstrating that upper limb motor nerve latency differed significantly between males and females. Although participants of both sexes had increased motor nerve latency, male participants had significantly greater latency than females.

In conclusion, the results provide evidence that carrying a backpack loaded to 30% bodyweight for 20 minutes has immediate effects on nerve conduction in the upper limb for both sexes. The quantitative comparisons clearly demonstrate that there are implications for the upper limbs caused by compression from the backpack shoulder straps. Specifically, short-term decreases in median nerve action potential amplitude and increases in latency are present even though these changes did not result in symptoms such as pain, tingling, or numbness in the hands. Nerve conduction deficits were consistent for male and female participants, although males had significantly greater motor nerve latency than females after backpack use. Increase in latency and decrease in amplitude of action potential in the median nerve after a short bout of walking could affect use of the thumb and finger muscles and consequently could affect manual task performance. It is also possible that the 20% decrease in amplitude of the sensory nerve response could result in reduced sensation on the fingers innervated by the median nerve. Reduced sensation on the lateral aspect of the palm could potentially impact tasks such as handling of syringes, medical equipment, tools, or ammunition. Movements of the first (thumb), second (index), and third finger could be slower to initiate because of increased latency of the median nerve response. Overall, the results emphasize the importance of studying the effects of compression of backpack straps on the upper limbs as this knowledge can be used to help maintain use of the thumb and fingers. Implications of this work are that the backpack should be dropped, if possible, before doing manual tasks or breaks should be taken to reduce the compression of the upper limb nerves. Also, the findings revealed that SMM was favorable to maintaining motor nerve conduction after backpack carriage while lower BMI was a risk factor for sensory deficit. In the long term, increased understanding of the effect of backpack straps on motor and sensory nerve conduction may allow for further guidelines to prevent injury and reduce incidents of backpack palsy. This first study measuring nerve conduction before and after backpack carriage adds to the current knowledge of how loads carried by backpack straps affect the upper limbs.

## Data Availability

The data that support the findings of this study are available upon request from the corresponding author.

## INSTITUTIONAL CLEARANCE

Institutional clearance approved.
